# Pazopanib and pancreatic toxicity: a case report

**DOI:** 10.1186/s13104-015-1154-4

**Published:** 2015-05-14

**Authors:** Marco Russano, Bruno Vincenzi, Olga Venditti, Loretta D’Onofrio, Raffaele Ratta, Francesco M. Guida, Giuseppe Tonini, Daniele Santini

**Affiliations:** Department of Medical Oncology, Campus Bio-Medico University of Rome, Via Alvaro del Portillo 200, 00128 Rome, Italy

**Keywords:** Pazopanib, Renal cell carcinoma, Pancreatic toxicity, Amylase, Lipase

## Abstract

**Background:**

Pazopanib is an oral multitargeted tyrosine-kinase inhibitor, used as a single agent to treat advanced renal cell carcinoma. Treatment with other tyrosine-kinase inhibitors is known to be associated with asymptomatic elevations of serum amylase and lipase levels. As regards the pazopanib, data are lacking in literature.

**Case presentation:**

We report one case of pancreatic toxicity associated with pazopanib administration. Before starting treatment, patient had no risk factors for pancreatitis. The patient, an Italian 68 years old woman, started pazopanib at doses of 800 mg daily as first-line therapy for metastatic renal cell carcinoma. Six months after the start of treatment, blood tests showed for the first time a significant increase in serum lipase and amylase in the absence of symptoms and radiological findings of pancreatitis. The patient continued treatment without interruptions or dose reductions. However, the continuation of the treatment led to a further increase of pancreatic enzymes. We tried to continue the treatment by reducing the dose but only the discontinuation was associated with normalization of amylase and lipase’s levels. On the other hand the treatment with pazopanib got prolonged response of the disease in the absence of signs of pancreatitis. We therefore decided to continue treatment with pazopanib 400 mg daily with close monitoring of blood levels of pancreatic enzymes.

**Conclusions:**

We hypothesize that the increase of pancreatic enzymes is not a dose-dependent event. The mechanism for pancreatic toxicity induced by tyrosine-kinase inhibitors is unknown and no predictive factors have been identified. There are no clear guidelines on the management of the drug in the presence of pancreatic enzyme increase. In any case, we believe that a careful monitoring of pancreatic enzymes during treatment with pazopanib is advisable.

## Background

Pazopanib is an oral multitargeted TKI (tyrosine-kinase inhibitor), used as a single agent to treat advanced renal cell carcinoma [1]. Treatment with other TKIs such as sorafenib and sunitinib is known to be associated with asymptomatic elevations of serum amylase and lipase levels, which may occur in up to 50 % of patients treated. Acute pancreatitis has been reported as a rare complication [2]. As regards the pazopanib, data are lacking: in a single-arm RCC (Renal Cell Carcinoma) trial, increases in lipase values were observed for 27 % (48/181) of patients. In the RCC trials of pazopanib, clinical pancreatitis was reported in <1 % (4/586) of patients [3].

## Case presentation

**Fig. 1:**
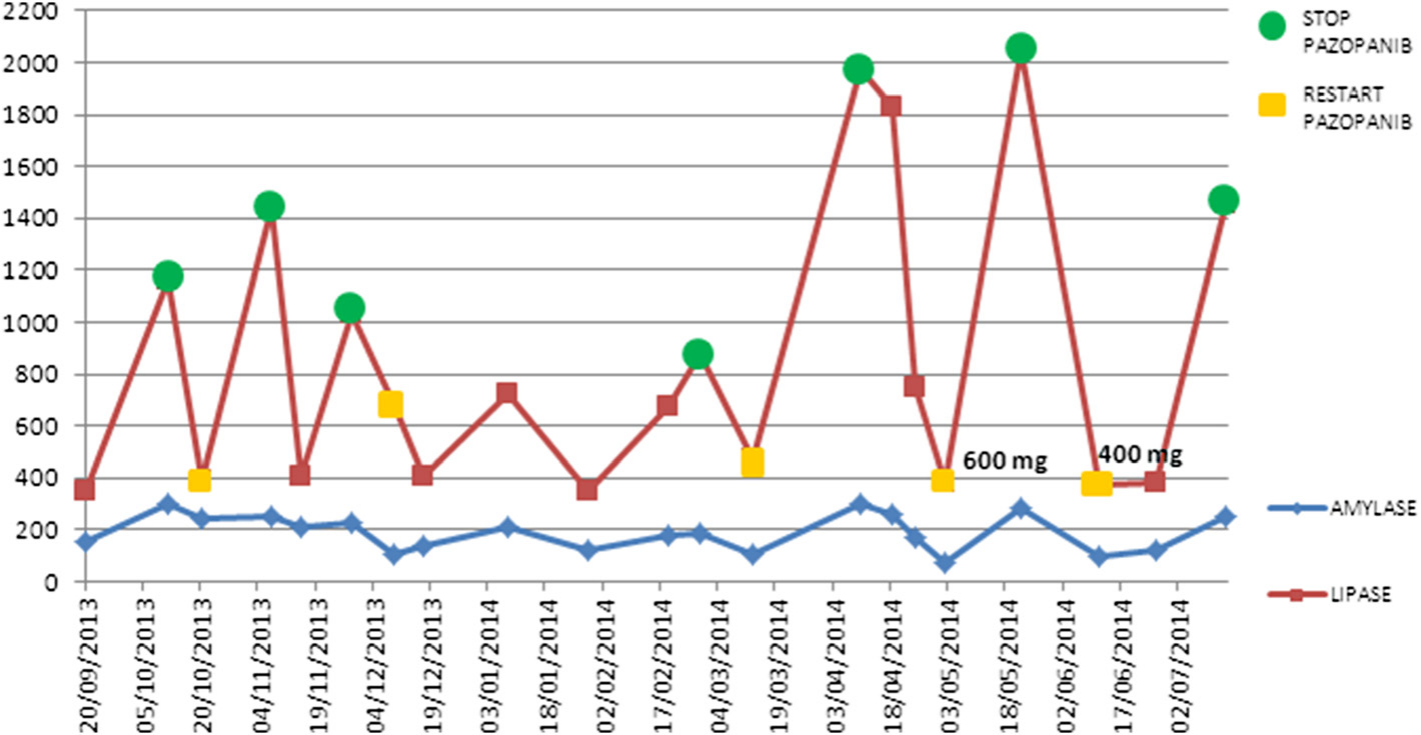
Correlation between amylase and lipase serum levels with pazopanib administration

We report one case of pancreatic toxicity associated with pazopanib administration. Before starting treatment, patient had no risk factors for pancreatitis: no history of alcoholism or smoking habit, cholelithiasis and biliary-pancreatic infections; we also excluded by MRI (Magnetic Resonance Imaging) or CT-SCAN (Computed Tomography-scan) the presence of cysts or pancreatic metastasis. This patient, an Italian 68 years old woman, started pazopanib (800 mg/die) as first-line therapy for renal cell carcinoma metastatic to liver and right and left adrenal glands, classified as favorable-risk according to MSKCC prognostic score (Memorial Sloan-Kettering Cancer Center score) [4]. The thorax CT scan and MRI of abdomen performed after 3 and 6 months of treatment showed liver and adrenal glands partial response. Six months after the start of treatment, blood tests showed for the first time an increase in serum lipase (2.5 × ULN − Upper Limit of Normal) and amylase (6 × ULN) in the absence of syntoms and MRI findings of pancreatitis. The patient continued treatment without interruptions or dose reductions. Blood tests showed a fluctuating trend of lipase and amylase (until 2 × ULN for lipase and 4 × ULN for amylase) until after about 18 months of treatment with stable disease, when it was documented a further significant increase in blood levels (lipase × 3 ULN and amylase × 5.5 ULN). Discontinuation of the drug led to a sharp reduction in these levels after only a week: serum lipase level was normalized, while serum amylase level was 4.5 × ULN. But the recovery of the treatment led to a persistent asymptomatic increase in lipase and amylase, therefore short interruptions of the drug were needed. This period of about 6 months was characterized by discontinuous treatment and parallel ups and downs of pancreatic enzymes. After 2 years and 3 months of treatment with stable disease, there was a dramatic increase in lipase (5 × ULN; amylase 5.5 × ULN) which led to discontinuation of the drug for almost a month before the levels for both pancreatic enzymes reach normal values. For this reason, the patient continued treatment with pazopanib at the dose of 600 mg/die. Nevertheless 3 weeks after resumption of treatment an increase in lipase even more significant was recorded: lipase 5 × ULN, amylase 5 × ULN. As a result of such events, after restoring the levels of lipase and amylase, we have decided to continue treatment with pazopanib but further reducing the doses to 400 mg/die. Four weeks after the start of this new treatment schedule amylase and lipase showed a new significant increase. We therefore decided to continue treatment with pazopanib 400 mg/die with close monitoring of blood levels of pancreatic enzymes. Throughout the course of treatment, no other significant alteration was found to blood tests about liver (transaminase and bilirubin levels) or kidney function (creatinine levels). After 2 months of intermittent pazopanib 400 mg/die, no clinical or radiological signs of pancreatitis appeared (Fig. 1). Despite repeated dose reductions and treatment interruptions pazopanib has so far achieved a prolonged disease control.

## Discussion

The mechanism for pancreatic toxicity induced by TKIs is unknown. No predictive factors have been identified. Hypothetical mechanism regarding acute pancreatitis induced by TKIs is the microvascular ischemia due to the anti-angiogenic effect that may predispose to pancreatic inflammation [5]. Other possible mechanism consist in the activation of pancreatic enzymes caused by the reflux of duodenal contents induced by decreased gastrointestinal motility. The increase of pancreatic enzymes could also be linked to apoptosis of acinar cells induced by inhibition of VEGF (Vascular Endothelial Growth Factor) [6]. Nevertheless specific data regarding pazopanib are lacking in literature. In the reported case, the increase of pancreatic enzymes did not appear to be a dose-dependent event.

## Conclusions

There are no clear guidelines on the management of the drug in the presence of pancreatic enzyme increase and no data regarding the real negative predictive role of enzyme increase in pancreatitis evolution. Further studies to identify the mechanisms of pancreatic toxicity and establish the management and the safety of treatment with pazopanib are needed. In any case, we believe that a careful monitoring of pancreatic enzymes during treatment with pazopanib is advisable. Hypothetically, the increase of pancreatic enzymes in the absence of obvious signs of pancreatitis may require changes to the schedule of treatment with pazopanib but not necessarily lead to a prolonged suspension of the treatment.

## Consent

Written informed consent was obtained from the patient for publication of this Case report and any accompanying images. A copy of the written consent is available for review by the Editor of this journal.

## Abbreviations

TKI: Tyrosine-kinase inhibitor
RCC: Renal cell carcinoma
MRI: Magnetic resonance imaging
CT-SCAN: Computed Tomography-scan
MSKCC prognostic score: Memorial Sloan-Kettering Cancer Center prognostic score
ULN: Upper Limit of Normal
VEGF: Vascular endothelial growth factor
